# Neural Mechanisms and Brain Alterations in Orofacial Pain: A Narrative Review of Human Neuroimaging

**DOI:** 10.3390/diagnostics16172851

**Published:** 2026-09-04

**Authors:** Nelson Freitas, Carlos Silva Faria, Daniel Humberto Pozza

**Affiliations:** 1 Unit of Experimental Biology, Department of Biomedicine, Faculty of Medicine, University of Porto, 4200-319 Porto, Portugal; 2 Department of Surgery and Physiology, Faculty of Medicine, University of Porto, 4200-319 Porto, Portugal; carlosafaria@gmail.com; 3 Institute for Research and Innovation in Health and IBMC (i3S), University of Porto, 4200-135 Porto, Portugal

**Keywords:** orofacial pain, temporomandibular disorders, trigeminal neuralgia, persistent dentoalveolar pain, magnetic resonance imaging, neuroimaging, brain connectivity, chronic pain, neuroplasticity

## Abstract

Orofacial pain is a complex and multifactorial condition that affects quality of life and presents important diagnostic and therapeutic challenges. Advances in neuroimaging techniques have enabled the investigation of nervous system alterations associated with pain perception and modulation. This review synthesizes current evidence on structural, functional, and neurochemical brain alterations identified through magnetic resonance imaging (MRI) in individuals with acute and chronic orofacial pain conditions. A comprehensive literature search of PubMed, Web of Science, and Scopus identified MRI studies (structural, diffusion, functional) in adults with temporomandibular disorders, trigeminal neuralgia, persistent dentoalveolar pain, or experimental pain. The main results demonstrated alterations along peripheral trigeminal pathways, brainstem nuclei, and central pain processing networks. Additionally, it was also possible to verify altered gray- and white matter integrity, causing disrupted connectivity within large-scale networks related to pain modulation and cognitive processing. While acute pain reflects transient activation of these pathways, chronic and neuropathic conditions can involve persistent structural and functional reorganization across sensory, affective, and cognitive networks. These findings support the involvement of distributed neural mechanisms and neuroplastic changes in many chronic orofacial pain conditions, while emphasizing that peripheral, central, psychosocial, and contextual factors may contribute to varying degrees across disorders and individuals. Multimodal MRI may provide a basis for future diagnostic, prognostic, and treatment response biomarkers, although most advanced MRI-derived markers remain investigational.

## 1. Introduction

Orofacial pain is a prevalent and clinically heterogeneous condition. It encompasses a broad spectrum of disorders involving the teeth, temporomandibular joints, masticatory muscles, cranial nerves, and associated structures. It may present as acute, subacute, chronic, episodic, continuous, localized, referred, or diffuse. Clinical presentations are often complex, and symptoms may not correlate clearly with identifiable structural pathology. In some cases, pain persists despite apparent tissue healing. In others, minimal clinical findings are accompanied by severe subjective distress. Referred pain, overlapping diagnoses, and comorbid headache or cervical disorders further complicate assessment. Chronic orofacial pain is particularly burdensome and is associated with peripheral and central sensitization, altered pain modulation, and psychosocial vulnerability and extends beyond physical discomfort, disrupting sleep, driving fatigue, and elevating stress reactivity. It impairs cognitive function and mental health, triggering attention issues, memory loss, anxiety, depression, and pain catastrophizing. Ultimately, these physical and emotional burdens restrict basic daily activities like eating and speaking, compromise work and social life, reduce overall quality of life, and increase healthcare utilization [1,2,3].

Temporomandibular disorders (TMDs) constitute the most common chronic orofacial pain condition, encompassing a broad spectrum of muscular and joint-related disorders that affect the masticatory system and the temporomandibular joints (TMJs). Clinically, TMD is characterized by regional facial and preauricular pain, functional limitation, and TMJ noises, often accompanied by impaired jaw mobility and difficulties in daily activities such as eating or speaking. Prevalence estimates range from 5 to 30% in adults, with higher rates in women and substantial impact on psychosocial functioning and quality of life. Chronic TMD may involve interactions among peripheral nociceptive mechanisms, altered nociceptive processing, pain modulation, psychological and sleep-related factors, and other biological vulnerabilities, with the relative contribution of these mechanisms varying among individuals, aligning the disorder with a biopsychosocial pain model. MRI and CBCT can improve anatomical characterization when imaging is clinically indicated, with MRI particularly useful for soft-tissue and intra-articular assessment and CBCT for osseous abnormalities. However, imaging should be used selectively and only when it will change clinical management. Overall, TMD represents a complex, multifactorial musculoskeletal and neurological pain disorder, with wide-ranging effects that extend beyond the TMJ to encompass systemic, behavioral, and cognitive domains [4,5,6,7]. TMD often follows a fluctuating clinical course in which symptoms remit in some individuals while emerging in others over relatively short periods, underscoring the dynamic nature of TMD manifestations and indicating that symptom persistence, spontaneous improvement, or recurrence may occur even without formal intervention, thereby complicating prognosis and treatment planning [8,9].

Trigeminal neuralgia (TN) is characterized by brief, unilateral paroxysms of severe, electric shock-like or stabbing pain. Innocuous triggers such as chewing, speaking, or light touch frequently precipitate attacks leading to decreased quality of life [10,11,12,13,14]. TN comprises classic (neurovascular compression), secondary (e.g., multiple sclerosis, tumors), and idiopathic forms [12,15,16]. Classical TN is commonly associated with clinically relevant neurovascular conflict involving the trigeminal root, particularly near the root entry zone, although neurovascular contact can also be found in asymptomatic individuals and is not by itself sufficient to establish causality. Arterial or venous contact is common, although tumors, cysts, or vascular malformations may also cause compression. Demyelination and other structural changes affecting the trigeminal root, which can contribute to abnormal excitability and ephaptic transmission. Alterations in voltage-gated sodium-channel expression and function have been proposed to contribute to neuronal hyperexcitability, although the specific molecular changes in human TN remain incompletely established. Additionally, electrophysiological and imaging studies demonstrate altered central nociceptive processing, amplified trigeminal transmission, and altered pain modulating networks. Together, these findings support a multi-mechanistic model in which peripheral demyelination, membrane hyperexcitability, and central network dysfunction interact to generate the characteristic paroxysmal phenotype [17,18,19,20].

Trigeminal nerve injury may contribute to peripheral hyperexcitability, while chronic pain may additionally be associated with altered thalamocortical processing [21,22]. Persistent abnormal afferent input may contribute to enhanced central nociceptive processing, through phenomena such as temporal summation and altered pain modulation [23,24]; thereby maintaining chronic pain and reflecting pain severity for the patient through generalized somatosensory processing dysfunction and its cognitive and emotional burden.

Dental pain activates pulpal nociceptors by inflammatory mediators generating sharp, well-localized pain through Aδ fibers, often followed by dull, throbbing pain mediated by C fibers as inflammation progresses and intrapulpal pressure increases. If untreated, pain can becomes more persistent, diffuse, and sometimes referred to adjacent structures. Due to the unique sensory organization of trigeminal innervation, orofacial pain can be diagnostically challenging, and although odontogenic pain represents most pain presentations in dental practice, the clinical picture may occasionally be ambiguous or mimic non-odontogenic conditions [25,26].

Importantly, other painful disorders, particularly TMD and neuropathic or musculoskeletal conditions, may exacerbate, coexist with, or even masquerade as toothache, complicating diagnosis and treatment planning. Effective management of odontogenic pain therefore requires not only elimination of the underlying source, but also careful pain control using both local and systemic strategies to prevent persistence of symptoms. Prolonged nociceptive input from dental tissues may contribute to peripheral and central changes in nociceptive processing and, in some individuals, to persistent post-treatment pain states, reinforcing the importance of early, accurate diagnosis and evidence-based pain management [25,26,27].

In parallel, experimentally induced orofacial pain models serve as essential tools for studying the early stages of trigeminal nociception and its modulation. These models are based on the activation of primary afferents and reproduce key biological processes such as peripheral sensitization, driven by inflammatory mediators that lower nociceptor thresholds. In this context, human functional imaging has provided preliminary insights into potential central correlates of trigeminal nociception [28,29,30,31,32,33], offering exploratory candidate markers of trigeminal nociceptive processing that might eventually inform future biomarker development. Functional MRI, using event-related designs adapted for safe trigeminal stimulation, demonstrates that intranasal delivery of controlled trigeminal nociceptive stimuli (e.g., ammonia gas) evokes robust Blood-Oxygen-Level-Dependent (BOLD) activations in recognized pain processing regions, including cortical and subcortical structures, as well as in brainstem regions. These responses confirm that fMRI can non-invasively capture activity within deep brainstem trigeminal pathways and may also reflect engagement of adjacent endogenous pain control regions such as the raphe nuclei. Together, these experimental and neuroimaging models provide an important and complementary framework for studying the human trigeminal pain system. This approach provides mechanistic insight into nociceptive processing and enabling the investigation of altered pain signaling in pathological conditions such as chronic TMD, trigeminal neuralgia, and primary headache disorders [23,31,32,33,34,35].

Across orofacial pain, peripheral sensitization may contribute to altered central nociceptive processing and, in susceptible individuals, to the persistence or amplification of pain. This is increasingly conceptualized as a maladaptive neurobiological state characterized by altered connectivity and plasticity in sensory, discriminative, and affective–cognitive circuits (e.g., thalamus and somatosensory cortex, limbic system including insula, anterior cingulate cortex, amygdala, among others) [36,37,38,39,40]. MRI techniques including structural MRI, diffusion imaging, BOLD-fMRI, and MR spectroscopy, enable non-invasive mapping of these brain and nerve changes and are increasingly important research tools for phenotyping and investigating potential prognostic biomarkers in chronic orofacial pain [2,35,41].

In this context, the objective of this review is to summarize and discuss the results from human studies using MRI techniques in acute induced pain, chronic TMD and neuropathic orofacial pain. For this purpose, a comprehensive literature search was conducted in January 2026 across PubMed, Web of Science, and Scopus, without temporal limits. The search strategy targeted human brain neuroimaging in orofacial pain using combinations of MeSH terms and keywords: “Brain”, “Facial Pain”, and “Magnetic Resonance Imaging” (including functional, structural, and diffusion-weighted MRI modalities). Inclusion criteria comprised adult populations presenting acute experimental pain or primary chronic orofacial pain conditions, specifically TMD, TN, or PIDP, and reported brain structural, diffusion, or functional connectivity outcomes. Observational studies and controlled trials were included. Exclusion criteria comprised non-human studies, participants under 18 years, case reports, narrative reviews, editorials, and studies not reporting MRI-derived brain outcomes. Two authors independently screened titles, abstracts, and full texts in blind mode using the Rayyan web application (https://www.rayyan.ai/), resolving discrepancies by consensus with a third author. Extracted data were organized into summary tables to map peripheral-to-central neural alterations across sensory, affective, and cognitive pain dimensions.

## 2. Neurobiology of Chronic Orofacial Pain

Chronic orofacial pain arises from complex biological interactions between peripheral and central nervous systems, and it is influenced by biopsychosocial factors. Conditions such as TMD, TN, burning mouth syndrome, and persistent idiopathic facial pain illustrate that peripheral pathology alone cannot account for symptom chronification, complicating diagnosis. Imaging studies further demonstrate that key regions such as the thalamus, a principal ascending relay, and the anterior cingulate cortex (ACC) contribute to affective–motivational and cognitive aspects of pain and participates, together with distributed prefrontal, brainstem, hypothalamic and midbrain systems, in pain modulation [21,42]. Collectively, these findings underscore that chronic orofacial pain reflects distributed nervous system dysfunction rather than purely local pathology [43,44,45].

At the peripheral and brainstem levels, chronic orofacial pain often involves sensitization of trigeminal primary afferents and second-order neurons within the trigeminal spinal nucleus, lowering activation thresholds and expanding receptive fields. These mechanisms underline clinical features such as allodynia, hyperalgesia, and referred pain patterns commonly observed in persistent facial pain conditions. Concurrently, the insular cortex, particularly its posterior subdivision, integrates nociceptive and interoceptive information and demonstrates spectroscopy-detectable neurochemical abnormalities, including altered glutamate, N-acetylaspartate, and choline levels, reflecting disrupted excitatory transmission and neuronal integrity [40,46]. Persistent nociceptive input may perpetuate altered central nociceptive processing even after the initial peripheral stimulus decreases, sustaining pain perception through maladaptive neuroplastic changes [38,47,48].

At supraspinal levels, chronic orofacial pain is characterized by structural and functional reorganization across networks mediating sensory, affective, and cognitive aspects of pain. Neuroimaging consistently implicates regions including the thalamus, primary and secondary somatosensory cortices (S1 and S2), anterior and mid-cingulate cortex, insula, prefrontal cortex, basal ganglia, medial temporal lobe structures, and primary motor cortex. Beyond nociceptive coding, large-scale networks such as the default mode network and prefrontal limbic circuits are frequently dysregulated, aligning with the substantial cognitive–emotional burden associated with chronic pain. MRI studies have reported alterations at multiple levels of the trigeminal sensory system, including the nerve, brainstem, thalamus, cortex and large-scale networks. However, these alterations are still under investigation. Cortical regions participating in lateral and medial pain systems, particularly the insula, anterior and mid-cingulate cortex, and prefrontal regulatory regions, show both structural and functional reorganization, while altered connectivity within descending modulatory circuits and corticostriatal motor networks suggests that persistent pain is associated with alterations in systems involved in affective regulation and motor control. These multi-level alterations collectively support a model in which chronic orofacial pain reflects maladaptive reorganization across the entire trigeminal sensory axis rather than isolated cortical dysfunction [36,49,50,51,52]. Alterations involving hippocampal and parahippocampal structures have been reported in some chronic orofacial pain cohorts and may relate to pain-related learning, stress and cognitive–affective processes [1,53,54].

Within TMD, the neurobiology is best understood through the integration of structural, functional, biomolecular, and psychosocial factors. The Diagnostic Criteria for TMD (DC/TMD) formalized a dual-axis model incorporating both physical diagnoses and psychosocial assessment, facilitating consistent classification and risk profiling [6]. Epidemiological data demonstrate marked sex dimorphism and multifactorial influences in TMD prevalence, with temporomandibular pain occurring predominantly in young and middle-aged adults and affecting women approximately twice as often as men, while factors such as age, tooth loss, parafunctional behaviors, psychosocial distress, and socioeconomic context further contribute to disease risk, symptom severity, and functional limitation [55,56,57,58].

Sustained nociceptive input from the masticatory muscles or temporomandibular joints promotes peripheral sensitization through inflammatory and ischemic mechanisms. Peripheral inflammatory and mechanical processes can alter the local release and signaling of mediators such as glutamate, substance P and CGRP, contributing to nociceptor sensitization and, in some contexts, neurogenic inflammatory responses. Ion channel remodeling, affecting TRPV1, acid sensing ion channels, purinergic receptors, and voltage-gated sodium channels, lowers activation thresholds and maintains nociceptor hyperexcitability, clinically manifesting as local hyperalgesia and reduced pressure pain thresholds. Persistent afferent input from these sensitized tissues can induce activity-dependent altered central nociceptive processing within trigeminal brainstem and thalamic–cortical pathways. This facilitates synaptic plasticity and amplification or persistence of nociceptive processing. Over time, these peripheral and central mechanisms contribute to pain persistence, spread of hypersensitivity, and transition from acute nociceptive signaling to chronic centrally maintained pain states [3,59,60,61]. Internal derangement represents the predominant TMJ pathology and can be confirmed with MRI, while additional but less frequent conditions include osteoarthritis, idiopathic condylar resorption, inflammatory arthropathies, trauma-related abnormalities, and tumor or tumor-like lesions that may clinically mimic disk displacement. Imaging and arthroscopic studies demonstrate that joint effusion correlates with greater disease severity. Synovitis and chondromalacia are closely associated with inflammatory progression and degenerative change underscoring the importance of early detection and targeted management in patients with inflammatory temporomandibular joint disease. These findings suggest that joint effusion may provide evidence of intra-articular inflammatory or pathological change, although its relationship with disease severity and pain is not sufficiently specific to serve as a standalone marker of advanced disease [62,63]. Biomarker research further indicates that inflammatory cytokines (IL-1β, IL-6, TNF-α, PGE_2_), matrix metalloproteinases, and oxidative stress markers contribute to peripheral sensitization and altered central nociceptive processing, while systemic and salivary markers correlate with pain intensity and functional limitation. Importantly, conservative therapies such as hyaluronic acid or platelet-rich plasma injections, low-level laser therapy, and stabilization splints can modulate inflammatory and oxidative biomarker profiles in parallel with clinical improvement, supporting an integrated mechanistic model of disease modulation [64]. Cohorts and longitudinal programs such as OPPERA further demonstrate that psychosocial stressors, sleep disturbance, and negative affect interact with biological drivers to influence TMD onset and persistence, reinforcing a biopsychosocial-biomolecular framework for chronic orofacial pain [7,8,65,66].

## 3. MRI as a Diagnostic Tool in Chronic Orofacial Pain

MRI has an important disorder-specific and adjuvant role in selected cases of chronic orofacial pain, particularly when structural, intracranial, neurovascular, or temporomandibular pathology is suspected. While clinical assessment remains foundational in orofacial pain disorders, MRI can serve as a valuable complementary tool, offering soft tissue contrast and multiplanar resolution that can reveal structural and neurovascular abnormalities. This capability is particularly valuable when the differential diagnosis includes intracranial lesions, trigeminal neurovascular contact, or some cases of TMD that influences diagnostic categorization and management planning [67,68]. In TMD management, MRI acts as an adjuvant reference modality for detailed visualization of TMJ soft tissues, such as articular disk position, joint effusion, and intra-articular changes, when advanced imaging is clinically indicated. Although the correlation between specific MRI findings and subjective pain intensity remains variable, identifying these anatomical alterations can help refine diagnostic categorization and inform targeted management strategies in select patients [69,70].

Beyond structural joint evaluation, advanced MRI techniques have expanded understanding of both peripheral and central mechanisms underlying chronic orofacial pain. Structural MRI studies have reported regional gray matter differences in several pain-related regions in chronic pain cohorts, although the direction, anatomical distribution and clinical correlates vary across disorders and studies, including reductions in somatosensory, prefrontal, cingulate, insular, hippocampal, thalamic, and cerebellar regions, alongside region-specific increases such as in the sensory thalamus, amygdala, basal ganglia, and periaqueductal gray [21,71]. In TN, gray matter volume and cortical thickness differences along trigeminal pathways and pain processing regions can be related to mechanisms that contribute to disease maintenance [2,72]. Diffusion MRI techniques, including diffusion tensor imaging and diffusion-weighted imaging, further characterize microstructural integrity of the trigeminal nerve and central white matter, detecting cisternal nerve alterations related to neurovascular compression and longitudinal remodeling after treatment, supporting their utility as potential imaging biomarkers [2,73].

Functional and metabolic MRI methods in TN further complement structural evaluation. Functional MRI demonstrates alterations in pain processing networks involving the anterior cingulate cortex, insula, somatosensory cortices, and thalamus, as well as dysconnectivity among major networks such as the default mode, salience, and sensorimotor systems. Resting-state approaches are particularly useful in TN because they enable phenotyping without requiring painful stimulation and may support prognostic exploration [40,42,74]. BOLD-fMRI offers non-invasive temporal sensitivity compared with positron emission tomography, although technical constraints related to facial stimulation and motion artifacts remain challenges in experimental design. These considerations are particularly relevant in orofacial pain studies, where facial movements and discomfort during scanning may further influence signal stability. Consequently, careful protocol design and advanced preprocessing pipelines are essential to ensure reliable interpretation of pain-related brain activity [35,75,76]. Additionally, MR spectroscopy provides complementary neurochemical information, with metabolites such as glutamate/Glx, N-acetylaspartate, and choline reflecting excitatory neurotransmission, neuronal integrity, and membrane turnover in pain-relevant cortices [40,46,77,78]. These approaches support the investigation of emerging imaging biomarkers for chronic orofacial pain conditions by experimentally demonstrating functional and structural alterations across key nociceptive and modulatory regions, including the thalamus, insula, primary and secondary somatosensory cortices (S1 and S2), anterior and mid-cingulate cortex, prefrontal cortex, basal ganglia, medial temporal lobe structures, and the primary motor cortex. Alterations within these interconnected networks reflect both abnormal sensory processing and maladaptive affective–cognitive modulation of pain, providing candidate features for future studies of disease stratification, prognosis and treatment response [43,79].

Despite its strengths, MRI has limitations. Variability in protocol selection, patient positioning, and interpretation criteria can influence diagnostic consistency, and not all chronic pain presentations correlate with identifiable structural abnormalities. Certain patients with predominantly muscular or myofascial pain may derive limited direct diagnostic benefit from MRI, underscoring the need for clinically guided imaging indications. Previous studies suggested that while MRI findings may correlate with specific symptoms such as joint noise and limited mandibular movement, associations with muscle pain can be weaker, reinforcing the importance of integrating imaging with comprehensive clinical evaluation [69,80,81]. In summary, MRI can be a key adjunctive diagnostic tool in selected chronic orofacial pain settings, especially in suspected secondary TN, neurovascular conflict, and complex TMJ pathology.

## 4. Experimentally Induced Orofacial Pain

**Figure 1:**
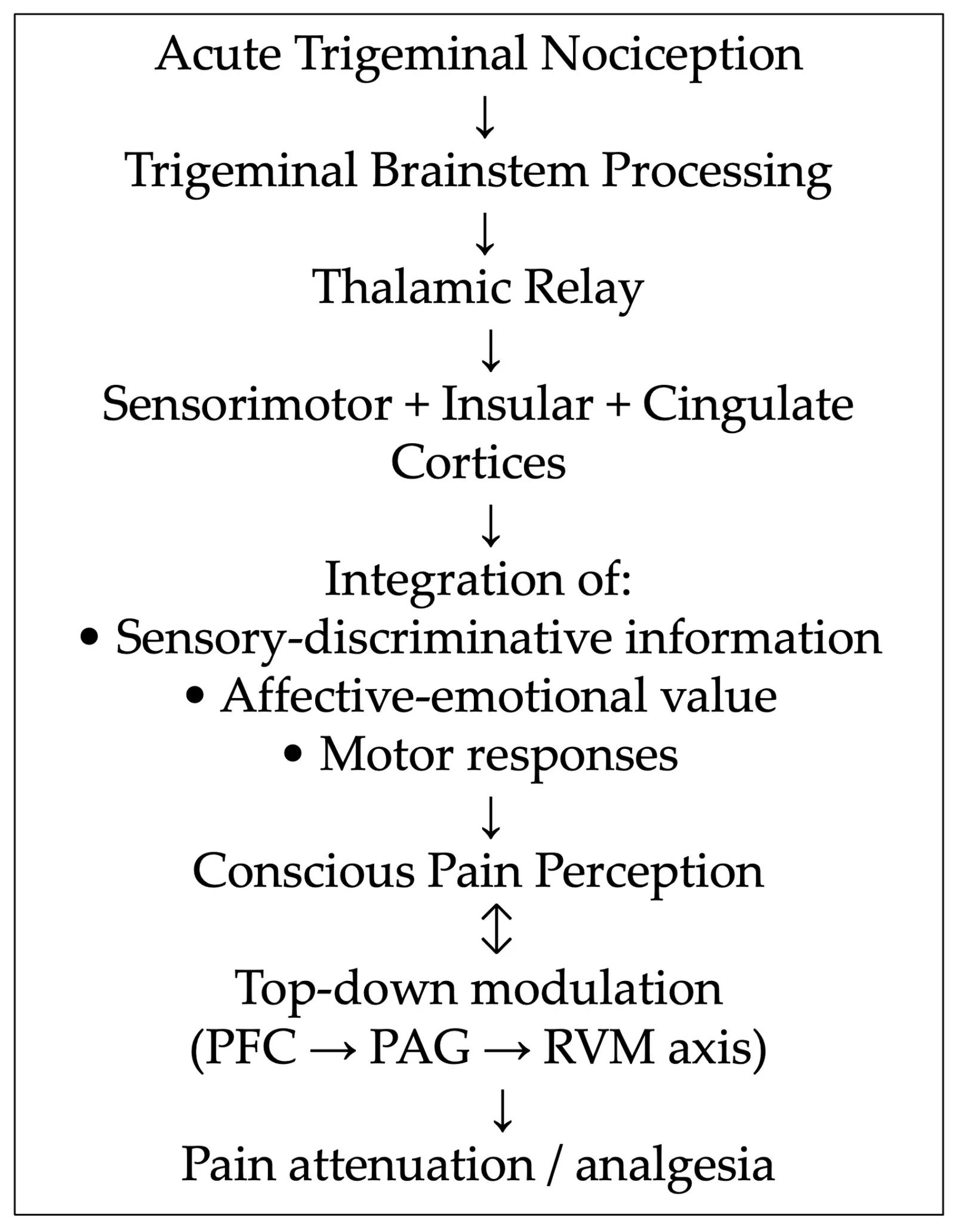
A simplified (not anatomically comprehensive) schematic representation of an ascending nociceptive pathway and descending modulatory loops during acute trigeminal nociception and conscious pain perception; not intended to represent all parallel, recurrent, or modulatory pathways. Legend—PAG: periaqueductal gray; PFC: prefrontal cortex; RVM: rostral ventromedial medulla. This figure was drawn utilizing Microsoft PowerPoint software (Microsoft Corporation, Redmond, WA, USA).

Acute trigeminal pain engages a distributed network of brain regions involved in nociceptive processing, sensorimotor integration, and cognitive–affective evaluation. Unlike chronic pain conditions, these responses largely reflect transient activation patterns along the trigeminal nociceptive pathway, extending from brainstem nuclei to cortical pain processing networks. A summary of human MRI studies evaluating acute orofacial pain is provided in Appendix A, Table A1. An ascending neuroaxis of acute orofacial pain, progressing from trigeminal brainstem relay to cortical integration across sensorimotor, insular, and cingulate networks, is illustrated in Figure 1.

Experimental models of orofacial pain provide a controlled framework for investigating the earliest stages of trigeminal nociceptive processing before the neuroplastic changes associated with chronic pain become established. Thermal, mechanical, electrical, and chemical stimulation paradigms combined with fMRI can be used to characterize the physiological processing of acute trigeminal nociception under controlled experimental conditions. Unlike chronic pain cohorts, these acute models minimize the influence of long-term sensitization, pharmacological treatments, and psychosocial comorbidities, thereby providing controlled models of physiological nociceptive processing and generating hypotheses about mechanisms that may also be relevant to chronic pain. Experimental paradigms establish the functional organization of nociceptive pathways that later become altered in disorders such as TMD and TN [31,32,35,43].

Despite differences in stimulation modality and anatomical target, acute orofacial pain consistently recruits a distributed network extending from the peripheral nervous system through the brainstem to higher cortical regions. Direct trigeminal stimulation may produce signal changes in the brainstem regions containing the trigeminal sensory nuclei and, depending on the imaging resolution and acquisition method, may also reveal signal changes near the cisternal trigeminal root and trigeminal ganglion. Functional connectivity studies further demonstrate coordinated interactions between the spinal trigeminal nucleus and subcortical relays, together with cerebellar connections to the insula, operculum, sensorimotor cortices, and frontal regions, highlighting the close integration of ascending nociceptive pathways with descending modulatory circuits [32,35].

At the cortical level, acute trigeminal nociception has been reported to activate regions responsible for sensory discrimination, affective processing, motor preparation, and cognitive evaluation of pain. Prominent activation was observed within the insular cortex, anterior and midcingulate cortices, primary and secondary somatosensory cortices, and primary motor cortex, reflecting the integration of nociceptive information with behavioral responses. Anterior cingulate cortex BOLD responses have shown correlations with subjective pain ratings, indicating a potential link between cortical activation and stimulus intensity [23,31].

Psychological factors can also influence these neural responses. Individuals with higher levels of pain catastrophizing exhibit greater activation within motor-related regions, including the primary motor cortex and cerebellum, accompanied by reduced activation of higher-order cognitive regions involved in pain modulation, such as the dorsolateral prefrontal cortex, anterior cingulate cortex, and insula. These findings support the concept that cognitive–emotional processes modulate nociceptive processing even during experimentally induced acute pain, reinforcing the interaction between sensory and psychological dimensions of pain perception [82].

Experimental tooth pain provides further evidence that nociceptive processing extends beyond classical sensory pathways. Increasing stimulus intensity produces greater activation of the anterior insula and anterior cingulate cortex, whereas lower-intensity stimulation preferentially recruits posterior cingulate regions. Simultaneously, activation of the facial representation within the primary motor cortex suggests immediate recruitment of protective motor responses involved in facial withdrawal and grimacing. Together, these observations suggest that acute dental pain rapidly integrates sensory encoding, emotional salience, and motor preparation following trigeminal afferent activation [31,83,84].

The spatial organization of nociceptive processing also depends on both the anatomical location and tissue origin of pain. Tooth pain preferentially activates the facial representation within the primary and secondary somatosensory cortices, whereas pain originating from the hand recruits more medial somatosensory and frontal cortical regions. Facial pain additionally elicits stronger activation of structures involved in emotional processing, autonomic regulation, and memory, including the hypothalamus, parahippocampal gyrus, precuneus, and amygdala. Differences have also been observed depending on the tissue stimulated, while muscle and cutaneous pain both engage a broad nociceptive network, they may produce subtle differences in activation patterns within trigeminal brainstem nuclei, supporting the existence of modality-specific processing within the trigeminal system [33,85].

Neuroimaging studies examining transitions between pain and analgesia further emphasize the dynamic organization of these networks. Pain states are characterized by increased activity within the thalamus, posterior insula, cingulate cortex, and frontoparietal regions, whereas analgesia is associated with greater recruitment of prefrontal cortical regions, cerebellum, and hippocampus, reflecting enhanced top–down modulation of nociceptive processing. Reduced posterior insular activity has been observed during experimentally induced dental pain relief and may represent one neural correlate of reduced nociceptive experience [34].

**Figure 2:**
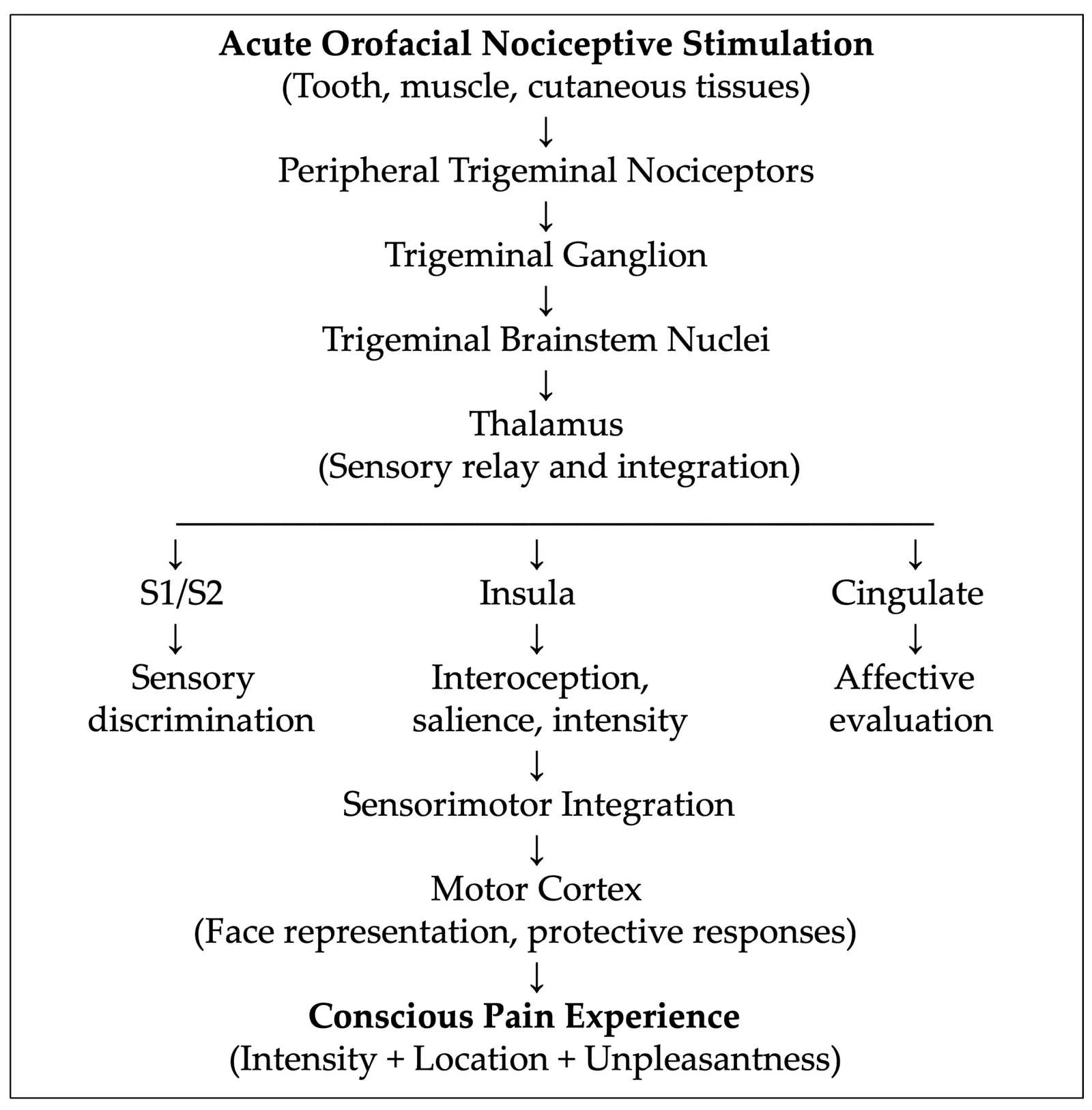
A brief schematic representation of the ascending neuroaxis during acute orofacial nociceptive stimulation, illustrating trigeminothalamic relay, thalamocortical divergence into sensory, insular, and cingulate networks, and subsequent sensorimotor integration shaping the conscious pain experience. Legend—S1/S2: primary/secondary somatosensory cortex. This figure was drawn utilizing Microsoft PowerPoint software (Microsoft Corporation).

As illustrated in Figure 2, acute experimental orofacial pain activates an integrated network extending from trigeminal brainstem nuclei to distributed cortical, cerebellar, and limbic regions. Rather than representing isolated areas of activation, these structures function as interconnected components responsible for the sensory-discriminative, affective, cognitive, and motor dimensions of pain. Consequently, experimental pain paradigms offer a valuable translational model that may help clarify potential functional changes observed in chronic orofacial pain conditions [43,49,50].

## 5. Neuropathic Orofacial Pain

Neuropathic orofacial pain conditions such as trigeminal neuralgia (TN) and persistent idiopathic dentoalveolar pain (PIDP) are associated with structural and functional alterations involving multiple levels of the trigeminal pain system, from peripheral nerve structures to brainstem, thalamic and cortical networks. A summary of human MRI studies evaluating neuropathic orofacial pain conditions is provided in Appendix A, Table A2.

Magnetic resonance imaging (MRI) has become a valuable tool for investigating the neural mechanisms underlying chronic orofacial pain by enabling the simultaneous assessment of structural, functional, and microstructural alterations throughout the trigeminal system. While conventional MRI remains fundamental for excluding secondary causes of facial pain and identifying peripheral abnormalities, advanced neuroimaging techniques, including structural MRI, fMRI, and diffusion MRI, have considerably expanded our understanding of the central nervous system adaptations associated with persistent pain. Unlike experimental pain paradigms, which characterize physiological nociceptive processing, MRI studies in patients with chronic orofacial pain reveal long-term neuroplastic changes involving both peripheral trigeminal pathways and distributed brain networks. These findings support the concept that chronic orofacial pain reflects dysfunction across multiple levels of the neuroaxis rather than a disorder confined to peripheral tissues [49,50,79].

Among patients with TN, structural MRI has substantially improved the characterization of neurovascular compression, particularly at the trigeminal nerve root entry zone, where chronic pulsatile contact is considered one of the principal mechanisms underlying classical disease. High-resolution MRI allows for visualization of vascular conflicts while simultaneously identifying morphological alterations affecting the trigeminal nerve, including changes in nerve volume, signal intensity, and root morphology. Beyond the peripheral lesion, structural neuroimaging has reported, with heterogeneous anatomical distribution and effect direction, gray matter alterations involving regions responsible for sensory, affective, and cognitive pain processing, including the thalamus, insula, anterior cingulate cortex, hippocampus, prefrontal cortex, amygdala, and primary somatosensory cortex. Importantly, several of these anatomical abnormalities appear to correlate with disease duration and pain chronicity, with some of these alterations being associated with disease duration; however, cross-sectional data cannot establish progressive remodeling or determine whether these changes are causes or consequences of chronic pain [1,2,72,86].

Functional MRI studies further suggest that chronic orofacial pain is accompanied by reorganization of cortical activation patterns and functional connectivity. Compared with healthy individuals, TN patients exhibit abnormal activity within the insular cortex, anterior cingulate cortex, sensorimotor cortices, prefrontal regions, cerebellum, hippocampus, and limbic structures, reflecting alterations in sensory discrimination, emotional processing, cognitive modulation, and motor integration. Resting-state investigations additionally reveal disruption of large-scale functional networks, including the default mode, salience, and sensorimotor networks, together with abnormal thalamocortical connectivity that appears to contribute to persistent pain perception and altered central nociceptive processing. Altered communication between the thalamus and cortical pain processing regions has emerged as a recurrent finding in TN, supporting the concept that dysfunctional thalamocortical circuits may contribute to persistent pain, although the extent to which these changes sustain symptoms independently of ongoing peripheral nociceptive input remains uncertain [21,42,51,87].

Diffusion MRI has provided complementary evidence by pointing toward microstructural alterations within both the trigeminal nerve and central white matter pathways. Diffusion tensor imaging is able to identify reduced fractional anisotropy and altered diffusivity within the trigeminal root, brainstem pathways, thalamic projections, corpus callosum, internal capsule, and limbic-associated white matter tracts, consistent with altered white matter microstructure, although diffusion metrics are biologically nonspecific and cannot by themselves establish axonal disruption. Similar abnormalities have also been observed within amygdala-related fiber pathways and cortical-brainstem connections, suggesting that chronic orofacial pain involves widespread reorganization extending beyond the primary trigeminal pathway. Collectively, these findings reinforce the concept that persistent pain is associated with distributed alterations across interconnected structural and functional networks [73,88,89,90].

Although neuroimaging abnormalities are most consistently described in TN, similar patterns of structural and functional reorganization have also been reported in TMD and other chronic orofacial pain conditions. Gray matter volume changes, altered somatosensory cortical organization, disrupted functional connectivity, and modifications within pain-related limbic networks indicate that different chronic orofacial pain disorders share common central mechanisms. These observations strengthen the hypothesis that prolonged nociceptive input promotes maladaptive neuroplasticity involving sensory, emotional, and cognitive pain processing systems. These findings are compatible with neuroplastic mechanisms that may contribute to symptom persistence in some patients, including after the initial peripheral trigger has resolved [50,71,91,92].

From a clinical perspective, MRI remains an established diagnostic modality in selected clinical settings, while quantitative structural, diffusion and functional MRI approaches are being investigated as potential biomarkers for disease characterization and treatment response. Although advanced MRI techniques are not yet routinely incorporated into clinical decision-making, continued development of quantitative imaging biomarkers, combined with emerging radiomic and artificial intelligence approaches, may improve patient stratification and support individualized therapeutic strategies for neuromodulation and surgical intervention in chronic orofacial pain [1,73,93,94,95].

**Figure 3:**
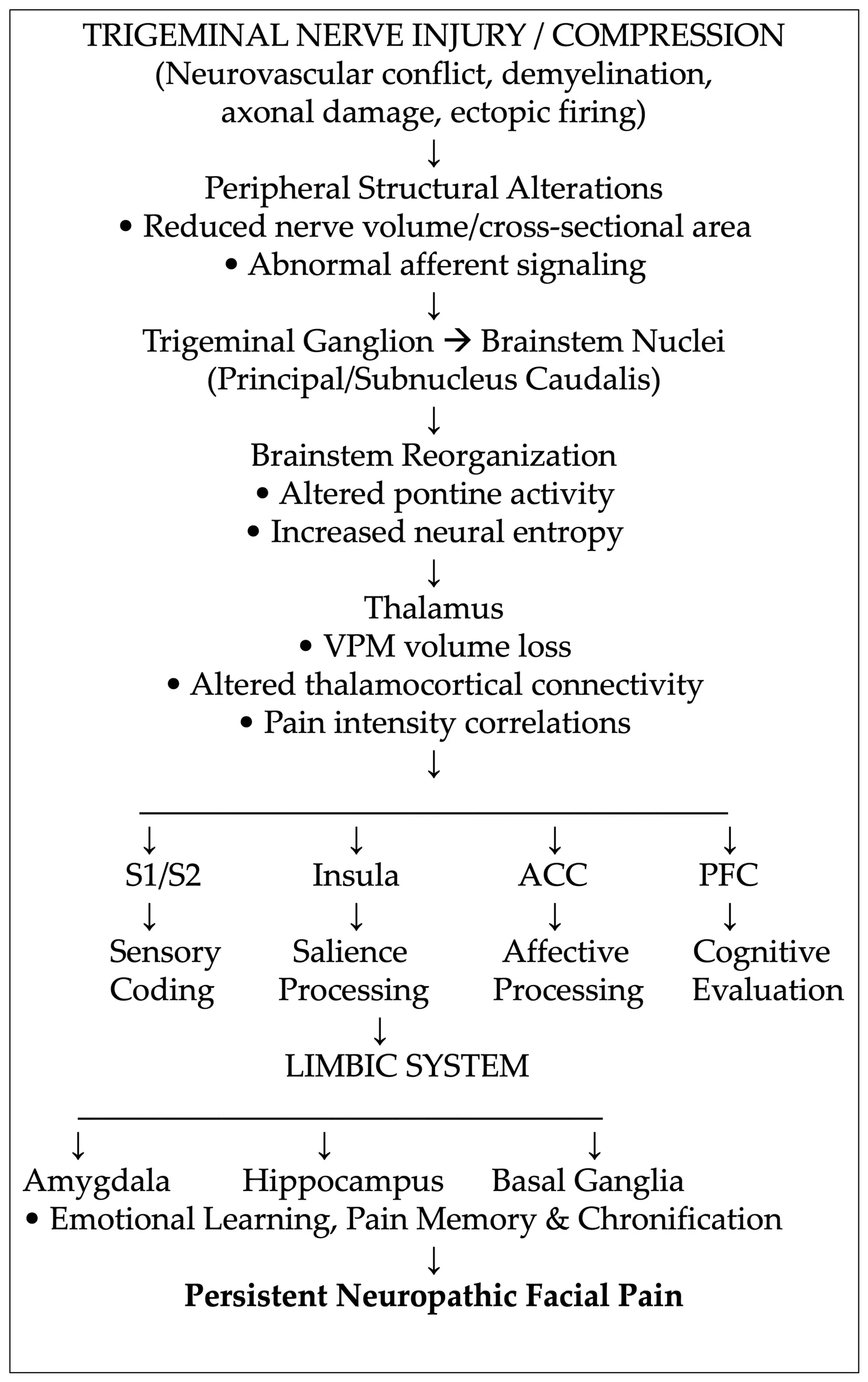
Conceptual model of associated peripheral and central alterations (putative functional roles). Directionality remains uncertain. Legend—ACC: anterior cingulate cortex; S1/S2: primary/secondary somatosensory cortex; PFC: prefrontal cortex; VPM: ventral posteromedial nucleus. Figure was drawn utilizing Microsoft PowerPoint software (Microsoft Corporation).

The mechanistic progression from localized neurovascular conflict and peripheral nerve damage to extensive central nervous system remodeling, including altered thalamocortical connectivity and limbic system recruitment, is conceptualized in Figure 3.

**Figure 4:**
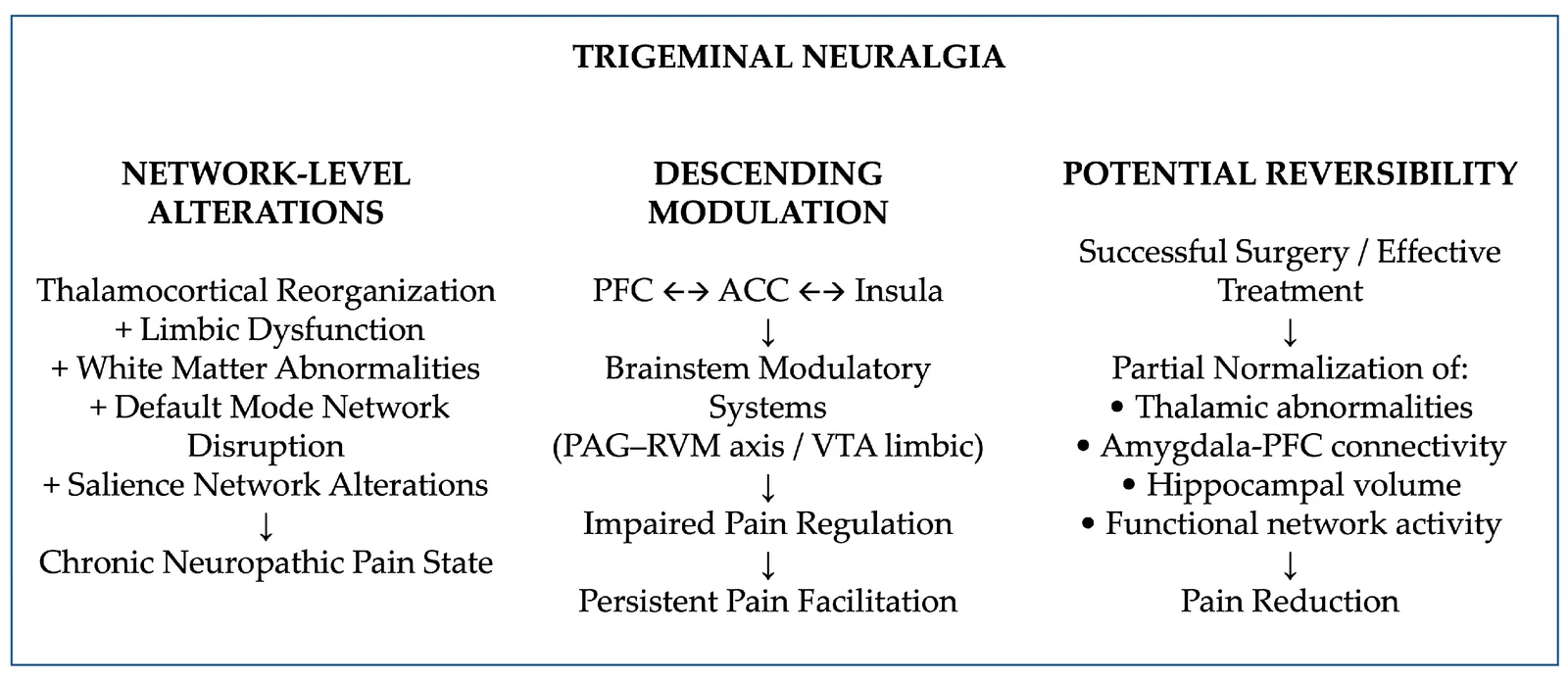
Schematic overview of central mechanisms in trigeminal neuralgia, main network-level alterations, descending pain modulation pathways, and structural/functional neuroplastic reversibility following partial treatment-associated changes reported in selected cohorts. Legend—ACC: anterior cingulate cortex; PAG: periaqueductal gray; RVM: rostral ventromedial medulla; VTA: ventral tegmental area. Figure was drawn utilizing Microsoft PowerPoint software (Microsoft Corporation).

The complex interplay between large-scale network disruption, altered descending modulatory control, and the potential for structural and functional normalization following intervention in trigeminal neuralgia is summarized in Figure 4.

## 6. Temporomandibular Disorders

Neurological alterations in TMD patients reflect changes in somatosensory processing and pain modulation networks, involving peripheral and central brain regions that contribute to both the sensory and affective dimensions of chronic pain. A summary of human MRI studies in TMD patients is provided in Appendix A, Table A3.

TMD comprise a heterogeneous group of musculoskeletal and articular conditions representing the most prevalent chronic orofacial pain condition. Although clinical manifestations range from localized joint dysfunction to persistent myofascial pain, increasing evidence indicates that TMD cannot be explained solely by peripheral pathology. Instead, chronic TMD pain results from the interaction of structural joint abnormalities, peripheral nociceptive input, psychosocial factors, and maladaptive neuroplastic changes within the central nervous system. Consequently, MRI has become an important tool not only for evaluating joint morphology but also for investigating the neural mechanisms underlying pain persistence and individual variability in symptom severity [5,50,96].

Structural MRI of the temporomandibular joint consistently demonstrates abnormalities involving disk displacement, joint effusion, synovitis, degenerative osseous remodeling, and condylar morphological alterations. Disk displacement, particularly when associated with inflammatory joint changes, represents one of the most frequent imaging findings and is commonly accompanied by increased synovial fluid accumulation, bone marrow changes, cartilage degeneration, and progressive remodeling of the mandibular condyle. Nevertheless, numerous studies have demonstrated only a modest correlation between structural abnormalities and pain intensity, indicating that peripheral imaging findings alone cannot fully explain symptom severity or chronicity. Despite the detailed structural insights provided by imaging, the diagnosis of TMD is fundamentally clinical. MRI is thus selectively utilized as a complementary tool, mainly in complex or atypical clinical scenarios where precise structural characterization is required. The incorporation of inflammatory biomarkers alongside MRI findings has further highlighted the contribution of local inflammatory processes to disease progression while emphasizing the multifactorial nature of pain generation in TMD [62,63,64,69].

Functional neuroimaging studies demonstrate that chronic TMD is associated with widespread alterations extending well beyond the temporomandibular joint itself. Compared with healthy individuals, some patients demonstrate abnormal activation of the insular cortex, anterior cingulate cortex, thalamus, prefrontal cortex, primary and secondary somatosensory cortices, and sensorimotor networks, reflecting disturbances in sensory discrimination, affective processing, motor control, and cognitive modulation of pain. Resting-state studies additionally demonstrate disruption of functional connectivity within the default mode, salience, and sensorimotor networks, consistent with altered central nociceptive processing and pain-related network reorganization. Like other chronic pain disorders, continuous pain and peripheral nerve signals can reorganize pain processing networks in the brain [36,41,49,50].

Diffusion MRI studies can provide complementary evidence of structural reorganization within the trigeminal pain system. Altered white matter integrity has been reported in pathways connecting the trigeminal nuclei, thalamus, sensorimotor cortices, corpus callosum, and limbic structures, suggesting impaired communication across networks involved in nociceptive transmission and pain modulation. Patients with chronic myofascial TMD can also exhibit regional gray matter alterations affecting the insula, anterior cingulate cortex, primary somatosensory cortex, and prefrontal regions, together with changes in cortical responses to somatosensory stimulation. These observations suggest that persistent myofascial pain is accompanied by widespread neuroplastic adaptations involving both structural and functional connectivity rather than isolated abnormalities confined to peripheral musculoskeletal tissues [50,71,91,92].

From a clinical perspective, MRI plays an increasingly important role in evaluating structural and neurobiological alterations, which may, in the future, help refine diagnostic assessment and inform potential individualized management strategies for TMD, although advanced MRI-derived markers currently remain primarily investigational. While TMD diagnosis remains primarily clinical, structural imaging facilitates the identification of intra-articular pathology in complex cases and assists in ruling out non-temporomandibular causes of orofacial pain. At the same time, advanced functional and diffusion imaging techniques can provide objective measures of neural structure, activity and connectivity that may help characterize altered central nociceptive processing and neuroplastic remodeling, offering potential biomarkers capable of explaining discrepancies between imaging findings and clinical symptoms. Although these techniques remain primarily research tools, the integration of multimodal MRI with clinical assessment, psychosocial evaluation, and emerging imaging biomarkers may ultimately support more personalized therapeutic strategies by identifying patients who are likely to benefit from conservative rehabilitation, pharmacological treatment, or targeted neuromodulatory interventions [68,79,95,96,97].

**Figure 5:**
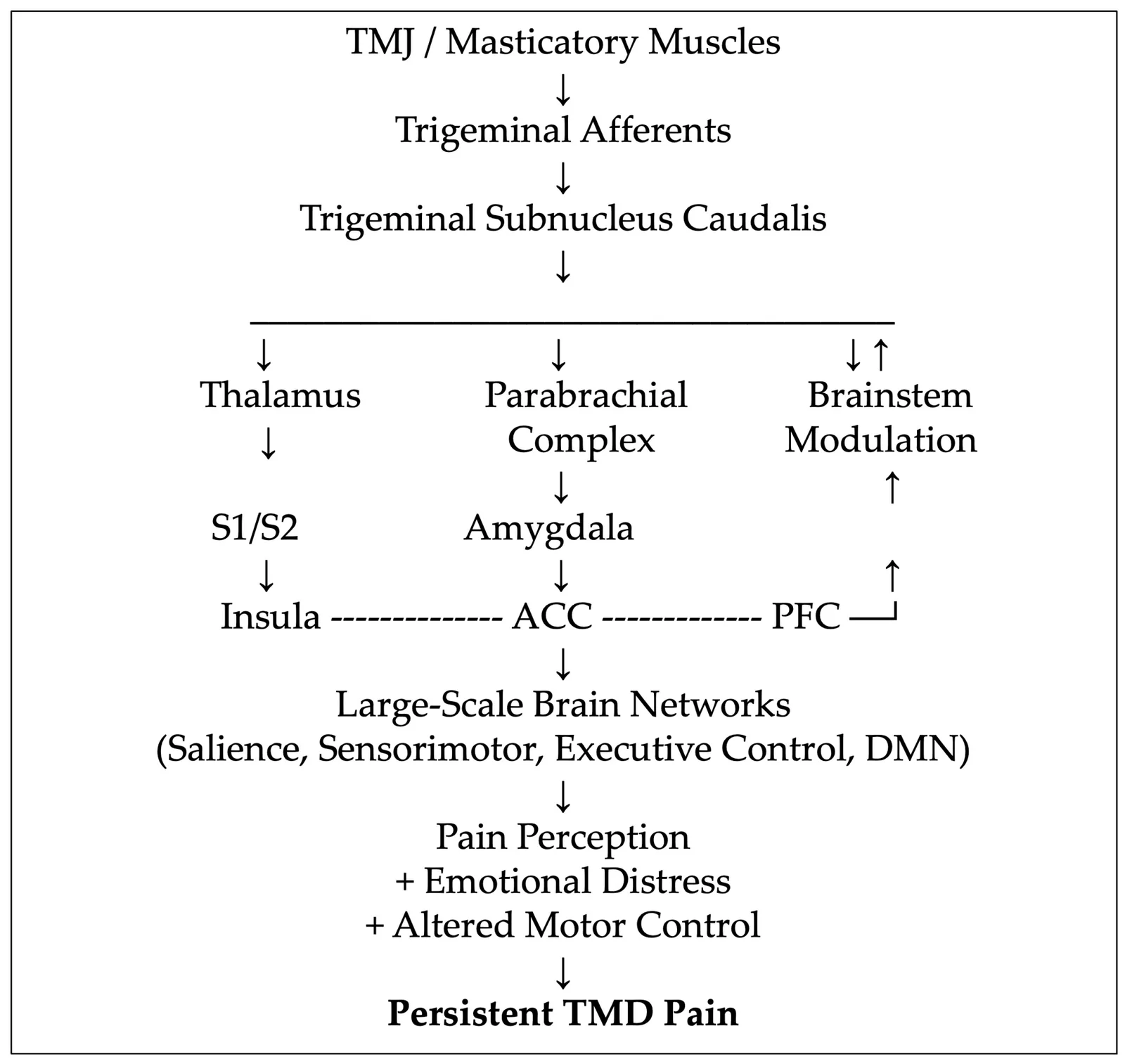
Conceptual schematic framework of ascending trigeminal pathways and large-scale network disruption in TMD, illustrating parallel thalamocortical and parabrachio-amygdalar processing loops contributing to chronic pain. Pathways are inferred from convergent neurobiology, not directly proven as TMD causal sequence. Legend—ACC: anterior cingulate cortex; DMN: default mode network; PFC: prefrontal cortex; S1/S2: primary/secondary somatosensory cortex; TMD: temporomandibular disorder; TMJ: temporomandibular joint. Figure was drawn utilizing Microsoft PowerPoint software (Microsoft Corporation).

As schematized in Figure 5, persistent TMD pain is driven by parallel ascending pathways, where sensory-discriminative thalamic routes operate in tandem with parabrachio-amygdalar loops to process affective distress and alter large-scale network dynamics.

The main structural, functional, and large-scale network alterations characteristic of the three orofacial pain states in human MRI studies described in this review are contrasted in Table 1.

## 7. Multimodal Integration and Clinical Implications

Integrating structural, diffusion, and functional MRI can improve mechanistic interpretation in research settings and may support future clinical stratification if validated. In TN, imaging can corroborate neurovascular conflict, characterize nerve microstructure, and reveal central network abnormalities that relate to symptom severity and treatment response [2,74]. Notably, although microvascular decompression often yields rapid relief, recurrence after treatment highlights the heterogeneity of TN and motivates investigation of peripheral and central factors that may influence treatment response [18,20]. Pharmacological management is first-line yet limited by side effects and variable long-term efficacy; percutaneous or radiosurgical options offer alternatives for refractory cases [93,98,99].

The processing of acute trigeminal pain involves complex, overlapping functional networks spanning from primary afferents and brainstem relays (including the parabrachial nucleus), together with descending pain-modulatory regions such as the PAG and rostral ventromedial medulla, to higher-order cortical centers (S1/S2, insula, ACC, PFC). This extensive multimodal integration demonstrates that sensory discrimination, emotional appraisal, and cognitive control are deeply interconnected from the earliest stages of nociception. From a clinical perspective, understanding these dynamic network interactions underscores why peripheral-targeted therapies may not fully address pain experience in all patients, especially where altered central nociceptive processing or psychosocial factors contribute to interindividual differences in persistence or recovery [30,32,35,100,101].

Neural responses are further influenced by the anatomical location, tissue origin, and psychological context of nociceptive stimulation. Experimental studies have demonstrated distinct somatotopic activation patterns for facial compared with extra-facial pain, while cutaneous and muscular nociceptive inputs recruit partially different brainstem and cortical circuits. Cognitive–emotional factors, particularly pain catastrophizing, also modulate cortical activation, reinforcing the concept that acute pain perception results from the interaction between peripheral sensory input and higher-order modulatory mechanisms rather than reflecting stimulus intensity alone [83,84,85,102,103].

Persistent trigeminal pain, particularly in TN, is accompanied by widespread structural and functional reorganization involving both brainstem and supraspinal networks. Neuroimaging studies consistently demonstrate alterations in functional connectivity within the periaqueductal gray matter, spinal trigeminal nucleus, thalamus, insula, anterior cingulate cortex, prefrontal cortex, hippocampus, and amygdala, indicating that chronic pain extends beyond peripheral nerve pathology to involve maladaptive neuroplasticity throughout distributed pain processing systems. These central changes are closely associated with mechanisms of altered central nociceptive processing, altered descending pain modulation, and neuroimmune activation, providing a biological explanation for pain persistence, amplification, and variability in treatment response [1,30,51,86,104].

Despite originating from distinct peripheral mechanisms, chronic TMD demonstrate remarkable neurobiological convergence with trigeminal neuropathic pain at the central level. Structural MRI markers of intra-articular pathology, such as disk displacement, joint inflammation, and osseous remodeling, must be contextualized within widespread functional alterations across the insula, anterior cingulate, sensorimotor, and prefrontal cortices [71,92]. This multimodal evidence highlights that structural damage alone poorly predicts pain severity. Instead, chronic TMD reflects a complex interplay of impaired descending pain modulation, altered sensorimotor integration, and altered central nociceptive processing [71,79,92,96,105]. Clinically, psychological factors like pain catastrophizing directly modulate these central networks, influencing jaw kinematics, motor behavior, and overall disability. Integrating structural and functional neuroimaging with comprehensive clinical and behavioral metrics is therefore essential for holistic diagnostic profiling and targeted therapeutic strategies [36,82].

Collectively, current evidence indicates that multimodal MRI provides a research framework for integrating structural, microstructural and functional measures across different orofacial pain phenotypes. Beyond providing structural insights, these imaging techniques hold potential to assist in patient stratification, prediction of treatment response, and longitudinal monitoring of disease progression. Future advances in imaging biomarkers, radiomics, and artificial intelligence are expected to further enhance individualized management by identifying clinically meaningful neuroimaging signatures capable of supporting precision medicine approaches across different chronic orofacial pain conditions.

## 8. Methodological Limitations and Diagnostic Challenges

Despite the promising results, this review has several limitations. From a methodological perspective, although a structured search strategy was performed, this is a narrative review. Consequently, the study selection process carries an inherent potential for selection and publication bias. Regarding the synthesized literature, the included studies vary in sample size, imaging modality, and analysis methods, limiting direct comparability. Second, heterogeneity in pain intensity, duration, and etiology may confound interpretation of neural alterations. Third, many studies are cross-sectional, preventing causal inference regarding the transition from acute to chronic pain. Finally, inconsistencies in task paradigms, stimulation types, and ROI definitions reduce the ability to generalize findings across studies. Future longitudinal, multimodal studies systematic reviews and meta-analysis are needed to overcome these limitations.

The clinical interpretation of MRI findings in chronic orofacial pain also presents important challenges. Structural abnormalities do not always correlate with symptoms, as neurovascular compression may be present in asymptomatic individuals, whereas some patients with trigeminal neuralgia show no clear evidence of compression. Similarly, structural alterations of the temporomandibular joint do not necessarily reflect pain severity or functional impairment, emphasizing the multifactorial nature of chronic orofacial pain [69,86]. Functional MRI is also limited by its indirect measurement of neuronal activity and is influenced by physiological noise, motion artifacts, and variability in acquisition and analysis protocols, reducing reproducibility across studies [35,75].

Clinical heterogeneity further complicates the identification of reliable neuroimaging biomarkers. Factors such as pain duration, psychological comorbidities, medication use, iatrogenic procedures, and overlapping neural alterations across different chronic pain disorders limit diagnostic specificity. Nevertheless, advances in multimodal MRI, radiomics, and artificial intelligence are expected to improve diagnostic accuracy, prognostic assessment, and patient stratification, supporting more personalized approaches to the management of chronic orofacial pain [94,95,97,106].

## 9. Future Directions

Future research should focus on developing robust and reproducible MRI biomarkers that integrate peripheral nerve abnormalities with central network alterations to improve diagnosis, patient stratification, prognosis, and treatment monitoring in chronic orofacial pain [2,74]. Future longitudinal multimodal studies are needed to characterize structural and functional plasticity before and after pharmacological, percutaneous, or surgical interventions, combining nerve-level diffusion imaging with brain structural and functional changes [73,87]. The integration of functional MRI with magnetic resonance spectroscopy may further clarify neurochemical alterations, including excitatory–inhibitory imbalance and neuroinflammatory processes within key pain processing regions such as the insula and anterior cingulate cortex [40,92].

Transdiagnostic comparisons between different chronic pain disorders, including TN, TMD, and migraine, may improve our understanding of shared and disease-specific mechanisms, particularly those involving altered central nociceptive processing and descending pain modulatory pathways [23,42,107]. Such approaches could help explain the variability in treatment response and support more personalized therapeutic strategies.

Despite these promising advances, most neuroimaging studies remain observational and methodologically heterogeneous, limiting their immediate clinical translation. Future efforts should therefore prioritize standardized acquisition protocols, longitudinal validation of imaging biomarkers, and the integration of clinical, imaging, and biomolecular data within multimodal predictive models.

Artificial intelligence, radiomics and machine learning methods may eventually improve differential diagnosis, treatment response prediction and risk stratification, but these applications require larger datasets, external validation, prospective evaluation and demonstration of clinical utility. Ultimately, the convergence of multimodal neuroimaging, imaging biomarkers, and artificial intelligence has the potential to transform the diagnosis and management of chronic orofacial pain, moving the field toward precision medicine and more mechanism-based, individualized patient care.

## 10. Conclusions

Orofacial pain arises from coordinated activity across a hierarchical network extending from peripheral trigeminal afferents and brainstem nociceptive nuclei to thalamic relays, cortical sensorimotor regions, and limbic systems, ultimately involving large-scale functional networks that shape perception, emotion, and behavior. Whereas acute orofacial pain primarily reflects transient activation within well-defined nociceptive pathways, chronic and neuropathic conditions produce persistent, multilayered structural and functional reorganization across sensory, affective, cognitive, and attentional systems. Although conditions such as temporomandibular disorders and trigeminal neuralgia differ in etiology and clinical presentation, both have been associated with alterations in some overlapping central pain processing systems, although the mechanisms, clinical phenotypes and degree of central involvement differ substantially.

These insights reinforce the growing recognition that chronic orofacial pain is best conceptualized as a heterogeneous condition in which peripheral pathology, central nociceptive processing, psychological factors and broader biological context may interact. Characterizing the hierarchical and network-level changes that underlie the transition from acute to chronic pain offers a path toward more accurate diagnostic markers, individualized interventions, and predictive models of treatment response. Future progress will depend on integrating longitudinal neuroimaging, standardized experimental paradigms, and advanced computational approaches capable of capturing the dynamic evolution of pain-related brain states.

Multimodal MRI has progressed substantially as a research platform and has established clinical roles in selected structural and neurovascular indications. Quantitative structural, diffusion and functional MRI approaches show promise as future diagnostic and prognostic biomarkers, but most remain insufficiently validated for routine clinical use. Its ability to detect network-level dysfunction, monitor treatment-related normalization, and contribute to individualized risk stratification may support a more integrated model in which peripheral pathology, central pain processing and psychosocial factors are considered together. This technological evolution lays the foundation for a more predictive, preventive, and personalized approach to one of the most challenging problems in pain medicine.

## Figures and Tables

**Table 1:** Brief Comparison of Central and Peripheral Mechanisms of Experimental Orofacial Pain, Temporomandibular Disorders, and Trigeminal Neuralgia.

Feature	Acute Orofacial Pain	TMD	Trigeminal Neuralgia
Dominant or proposed mechanisms	Physiological nociception	Heterogeneous: predominantly nociceptive, nociplastic in some individuals	Neuropathic mechanism; central pain processing alterations may coexist
Peripheral driver	Controlled stimulation of healthy tissues	TMJ/muscle	Trigeminal nerve injury/compression/other mechanisms
Brainstem changes	Transient activation	Reported brainstem sensitization and plasticity related alterations	Structural and functional alterations
Thalamic involvement	Physiological thalamic engagement	Variable thalamic involvement reported	Reported thalamic network changes
Cortical changes	Reversible activation	Reported structural and functional alterations	Reported structural and functional alterations
Limbic involvement	Acute affective processing	Reported limbic network alterations	Reported limbic network alterations
Large-scale networks	Temporary recruitment	Associated network connectivity alterations	Reported thalamocortical and network connectivity changes
Reversibility	Immediate	Observed post-treatment changes	Post-treatment alterations reported in some cohorts

Legend: TMD: temporomandibular disorder; TMJ: temporomandibular joint; TN: trigeminal neuralgia.

## Data Availability

No new data were created or analyzed in this study.

## Abbreviations

The following abbreviations are used in this manuscript:

Aδ	A-delta fibers
BOLD	Blood-Oxygen-Level-Dependent
CBCT	Cone-Beam Computed Tomography
CNS	Central Nervous System
DC/TMD	Diagnostic Criteria for Temporomandibular Disorders
fMRI	Functional Magnetic Resonance Imaging
MRI	Magnetic Resonance Imaging
PIDP	Persistent Idiopathic Dentoalveolar Pain
RCT	Randomized Controlled Trial
TMD	Temporomandibular Disorders
TMJ	Temporomandibular Joint
TN	Trigeminal Neuralgia
V1	Ophthalmic division of trigeminal nerve
V2	Maxillary division of trigeminal nerve
V3	Mandibular division of trigeminal nerve

## Appendix A

**Table A1:** Included Human MRI Studies of Experimentally Induced Acute Orofacial Pain.

Reference	Participants	Intervention/Pain	Image	Main MRI Findings
[102] Non-RCT	16 healthy, 7F, 25.8 ± 1.4 y	Pain intensity: Lip 5.7 ± 0.3, Cheek 5.6 ± 0.5, Thumb 5.8 ± 0.3, Toe 3.6 ± 0.3	Structural MRI Functional MRI	Stronger mPFC/OFC, PVT, hypothalamus, lPB; lPB/SN ↓ for face, ↑/minimal for body; hypothalamus ↓ both (face > body); posterior PVT ↓ bilateral face, little change body; amygdala/BNST no differences
[33] Non-RCT	23 right-handed, 12F, 27.8 ± 4.2 y	Hand vs. Face electrical pain + recognition task without pain	Structural MRI Functional MRI	Face vs. hand pain memory: stronger in right parahippocampus & precuneus; recognition confidence linked to bilateral fusiform, left amygdala & parahippocampus; face fear correlated with ↑ left thalamus activity
[82] Non-RCT	34 healthy: 17, 11F, 26.2 ± 2.3 y vs. 19, 7F, 34.7 ± 2.4 y; 2 in both studies	Acute masseter pain averaging at 4.7 ± 0.2 (study 1) and 5.6 ± 0.2 (study 2)	Structural MRI Functional MRI	Resting pain ↑ CBF in orofacial S1/S2, bilateral DLPFC, ACC, insula, hippocampus, cerebellum (higher PCS); Chewing pain: PCS associated with ↑ activity in S1/M1, thalamus, cerebellum, trigeminal motor nucleus and ↓ activity in DLPFC, ACC, posterior insula; higher PCS linked to ↑ motor and ↓ cognitive/integrative activity
[34] Non-RCT	14 healthy right-handed, 25.1 ± 4.5 y	Acute electrical dental pain; intensity set to NRS 5/10; pain offset after anesthesia 2.8 ± 3.73 min	Structural MRI Functional MRI	Pain ↑ L thalamus, L posterior insula, R ACC/PCC, R inferior parietal, R STG, L visual cortex, R vlPFC; Analgesia: ↑ L thalamus, R inferior parietal, L visual cortex, bilateral DLPFC, R vlPFC, L cerebellum, R hippocampus; Pain → Analgesia: ↓ L posterior insula; Analgesia → Pain: no significant change
[31] Non-RCT	13 healthy right-handed males, age 22–49 (mean 33.6)	Dental pain induced by electrical stimulation	Structural MRI Functional MRI	Stimulus strength → ↑ BOLD in bilateral anterior insula (aIC) and L pregenual ACC (pgACC); widespread bilateral activation across sensory-motor, frontal, temporal, parietal, occipital, cerebellar, cingulate/insular, and subcortical regions (thalamus, putamen, brainstem)
[84] Non-RCT	30 healthy: 15 subcutaneous, 17 intramuscular	Maximum pain scores SC and IM hypertonic saline injection: 4.73 ± 0.51 and 4.35 ± 0.56	Structural MRI Functional MRI	Both: prolonged ↓ BOLD in contralateral M1 (≥6 min, persisting after pain); muscle pain only → brief early M1 ↑ (brainstem motoneuron region); no ipsilateral M1 change or overall modality difference; both activated thalamus, S1/S2, midcingulate cortex, insula, and cerebellum.
[85] Non-RCT	30 healthy, 8F, 19–52 y	Rapid-onset acute orofacial pain; max pain scores: SC 4.73 ± 0.51, IM 4.35 ± 0.56	Structural MRI Functional MRI	SpVo: ↑ for muscle & cutaneous pain; persists post-pain. SpVi: ↑ only for cutaneous pain. SpVc: ↑ for both pain types. Vp: ↑ only for muscle pain; no effect from isotonic saline. Medulla/raphe: ↑ for both pain types.
[83] Non-RCT	8 healthy, 4F, 21–28 y	Electrical stimulation acute tooth pulp pain	Structural MRI Functional MRI	S1/S2: Tooth pain ↑ in lateral/anterior S2 + ipsilateral face S1; Hand pain ↑ in medial/posterior S2 + contralateral S1. IC: Bilateral activation; strongest intensity coding in left medial/posterior IC Left ACC: ↑ strong tooth pain; right posterior ACC: ↑ weak tooth pain. M1: Tooth pain ↑ left ipsilateral face area Frontal cortex: Tooth pain ↑ medial/inferior frontal gyri; Hand pain ↑ superior frontal & orbital cortex. Anterior left insula: ↑ tooth pain but no intensity coding; Right precentral face area: intensity coding only.
[23] Non-RCT	11 healthy, 3F, 23.3 ± 2.0 y	Electrical stimulation acute Trigeminal pain	Structural MRI Functional MRI	pACC: Hemodynamic responses increase linearly with individual pain ratings. Peak activation (train > single pulse): pACC, brainstem, contralateral hippocampus.
[32] Non-RCT	54 healthy, 31F, 26.0 ± 3.9 y; 18 controls, 16F, 31.5 ± 11.1 y.	Chemosensory acute trigeminal pain	Structural MRI Functional MRI	Nociceptive input: clusters in cerebellum & PAG. Left spinal trigeminal nucleus: cerebellar lobules, rostral pons, bilateral thalamus, PAG, red nucleus, nucleus cuneiformis. Cerebellar–cortical connectivity: Vermis VIIIa, L VI & posterior VIIIa: ↑ links with putamen, insula, operculum, supramarginal, precentral & lingual gyri.
[35] Non-RCT	23 healthy, 11F, 28 yr, (range 20–39)	Chemosensory acute trigeminal pain: Ammonia = 5.78 ± 2.23; Odor stimuli = 3.56 ± 1.87.	Structural MRI Functional MRI	Bilateral: Insula, thalamus, mid-cingulate, amygdala, precentral gyrus, calcarine, cerebellum, MTG, brainstem. Ipsilateral: Caudate, putamen, supramarginal & precentral gyri, pallidum. Contralateral: Left ACC, postcentral & middle frontal gyri, pallidum. Trigeminal input: Activation at pons nerve entry zone + ipsilateral trigeminal ganglion. Refined localization: Reduced smoothing → pons/midbrain signals likely substantia nigra & nucleus ruber

Legend: ACC: anterior cingulate cortex; BNST: bed nucleus of the stria terminalis; BOLD: blood-oxygen-level-dependent contrast; CBF: cerebral blood flow; DLPFC: dorsolateral prefrontal cortex; fMRI: functional MRI; IC: insular cortex; L/R: left/right; lPB: lateral parabrachial nucleus; M/F: male/female; M1: primary motor cortex; mPFC: medial prefrontal cortex; MRI: magnetic resonance imaging; MTG: middle temporal gyrus; n: number of participants; NRS: numerical rating scale; OFC: orbitofrontal cortex; PAG: periaqueductal gray; PCC: posterior cingulate cortex; PCS: Pain Catastrophizing Scale; pgACC: pregenual anterior cingulate cortex; PVT: paraventricular thalamus; S1/S2: primary/secondary somatosensory cortex; SN: substantia nigra; SpVc/SpVi/SpVo: spinal trigeminal nuclei (caudalis/interpolaris/oralis); STG: superior temporal gyrus; TG: trigeminal ganglion; ↑/↓: increase/decrease; Vp: trigeminal principal sensory nucleus; vlPFC: ventrolateral prefrontal cortex; y: years.

**Table A2:** Included Human MRI Studies of Trigeminal Neuropathic Pain and other Orofacial Pain Conditions.

Reference	Participants	Intervention/Pain	Image	Main MRI Findings
[72] Case–control observational study	81 TN, 62%F, 27–83 y, MMSE > 24, right-handed. 30 healthy, 57% F, 41–74 y	Primary TN: duration 8 y; pain 5/10; BNIPS: 93.5% Grades IV–V/Vascular decompression, Radiofrequency	Structural MRI	Intralaminar, ventral, and associative nuclei show GMV reductions with some lateralization (right VPL/VM, bilateral LGN, right PuA/M). 6-mo post surgery: partial normalization, some differences NS. Pain intensity negatively correlated with right VPL/VM
[1] Cohort observational study	61 TN, 36F, 64.9 ± 12.0 y; 61 controls 36F, 64.8 ± 12.1 y	Pain: 8.98/10, BNI: I–V; duration 7 y/Gama Knife Radiosurgery	Structural MRI	Hippocampus (TN): Baseline bilateral CA4, DG, ML HP volume loss; Post-surgery responders: bilateral increases in CA1–4, DG, ML HP, subiculum; normalization to HCs in CA4, DG, ML HP; females showed greater ipsilateral increase.
[21] Cohort observational study	25 TN, 15F, 58.7 ± 6 y; 20 controls, 13F, 55.7 ± 7.8 y	Pain 9.3 ± 0.7 pre-surgery, 3.0 ± 2.9 follow-up; duration 85.7 ± 86.1 months; pain location V1/V2/V3: 16/76/76%/Radiofrequency rhizotomy	Structural MRI Functional MRI	TN network-level: Baseline reduced PC in SMN & DMN vs. HCs; Post-surgery PC normalized across modules; subcortical PC correlated with pain duration; higher pre-surgical DMN connectivity predicted better response.
[36] Case–control observational study	43 TN, 32F, 47.3 ± 2.3 y; 57 controls, 35F, 43.4 ± 1.7 y; 16 tonic pain, 5F, 24.9 ± 1.2 y	PTTN pain: 3.8/10, duration 6.0 ± 0.9 y; Tonic pain controls: 20 min induced orofacial pain, maintained at 5/10	Structural MRI Functional MRI	Chronic pain: Baseline reduced resting-state power & PCC connectivity in DMN (precuneus, PCC, bilateral IPC, mPFC) vs. HCs; ISOs reduced in DMN for both chronic pain & tonic pain controls; no medication effects on power
[108] Case–control observational study	26 PIDP, 11F, 54.7 ± 9.7 y; 13 controls 11F, 53.3 ± 10.6 y	PIDP: mean duration 8.0 ± 6.5 y (0.6–20); mean intensity 55.5 ± 25.6/100 (SF-MPQ VAS & CPI)/dentoalveolar stimulation	Structural MRI Diffusion MRI Functional MRI	PIDP: ↑ activation vs. HCs in S1, S2, IPL, insula, premotor & prefrontal cortices, thalamus; strongest during stimulus intensity matching
[109] Case–control observational study	18 toothaches, 10F; 18 matched controls	Pain 6.36 ± 1.49, duration 2.04 ± 1.08 y/None	Structural MRI Functional MRI	TA baseline: ↑ DC & FC in RLG, LMTG, RPG; RLG DC-HADS, LMTG DC-VAS; ROC analysis high better diagnosis
[88] Case–control observational study	9 TN (7F, 64.9 ± 2.6 y); 18 neuropathy, 15F, 48.0 ± 1.7 y; 20 TMD, 16F, 45.7 ± 2.9 y; 26 controls 21F, 52.3 ± 2.95 y	TN pain: 3.2 ± 0.8, 14.6 ± 4.3 y. Neuropathy pain: 3.9 ± 0.4, 4.8 ± 0.8 y. TMD pain: 3.7 ± 0.5, 9.1 ± 2.0 y/None	Structural MRI Diffusion MRI	TN (pain-side nerve): 47% decrease in total nerve volume and 32% decrease in maximal cross-sectional area vs. controls
[76] RCT	30 TN, 17F, 50.6 ± 12.1 y (conventional RF), 47.9 ± 9.9 y (pulsed RF)	TN Pain 8/10, 3.87 y (CRF), 3.47 y (PRF)	Structural MRI Functional MRI	CRF & PRF ↓ VAS vs. pre; CRF greater ↓ at 3 & 6 mo. fMRI pre-ablation: S2, angular gyrus, frontal cortex, parahippocampus, insula, ACC, thalamus, putamen, caudate, ventral diencephalon; 1 mo post: S1, angular gyrus, frontal cortices, anterior insula, ACC; post-ablation: ↓ BOLD in S2, frontal operculum, parahippocampus, posterior insula, basal ganglia, thalamus, ventral diencephalon
[42] Cross-sectional observational study	39 TN patients	Chronic Trigeminal Neuralgia pain/None	Structural MRI Functional MRI	TN causal connectivity: Thalamus → dACC pain scores; pain matrix interactions: Caudate → S1, Thalamus → S1, Insula → Thalamus, S1 → Insula/S1 → Thalamus pain; additional network: Caudate, Precentral, Supramarginal, Bankssts → ITG pain scores
[107] Case–control observational study	21 TN, 14F, 50.6 ± 8.0 y; 33 controls, 28F, 45.2 ± 9.7 y	TN/None	Structural MRI Functional MRI	TN: Reduced ALFF in dorsal attentional network and lower ReHo. Increased connectivity between somatomotor and dorsal attentional networks.
[74] Cross-sectional observational study	39 TN patients	Chronic Trigeminal Neuralgia pain/None	Structural MRI Functional MRI	Deep learning (CNN & GCNN): decoded low vs. high pain (dACC, fusiform, insula, precentral); predictions reported pain, generalized across sex & surgery; key pain regions: superior temporal, insula, fusiform, precentral, superior frontal, supramarginal, plus 17 others (dACC, thalamus).
[22] Case–control observational study	76 CTN, 52F, 56 ± 13 y; 72 controls, 47F, 54.5 ± 14 y	Chronic Trigeminal Neuralgia pain/None	Structural MRI Functional MRI	Brain entropy changes: Increased in thalamus and pons, decreased in inferior semilunar lobule; low positive correlation with neuropsychological scores.
[93] Cohort observational study	22 TN, 12F, 56.5 ± 10.9 y); 19 controls, 10F, 55.4 ± 9.3 y	TN Pain: 72.8 ± 27.5 mm, 6.2 ± 4.8 y/Microvascular, Balloon Decompression	Structural MRI Functional MRI	TN: ↑ connectivity R insula, bilateral thalamus; Non-responders: ↑ hippocampus–hippocampus, ↓ ACC, L amygdala, R hippocampus; TN duration: ACC–amygdala/ACC–R hippocampus (negatively).
[86] Case–control observational study	60 TN, 36F, 62 ± 13.2 y; 49 controls, 28F, 61.8 ± 9 y	TN pain: 7.7 ± 1.8/None	Structural MRI	Reduced GM in S1, S2, OFC, ACC, insula, thalamus, putamen, caudate, DLPFC, precuneus, and cerebellum. Longer duration associated with lower GM in rACC, parahippocampus, contralateral MTG, and ipsilateral right ITG
[2] Cross-sectional observational study	20 TN; 21 matched controls	Chronic Trigeminal Neuralgia pain/None	Structural MRI Diffusion MRI Functional MRI	TN: Insula ↓LGI (L), ↓ cortical thickness, surface area unchanged; WM (FA): ↓ L external capsule, superior corona radiata, posterior limb internal capsule, FA–insular LGI; FC: ↑ insula–PCC & thalamus
[51] Case–control observational study	29 TN, 19F, 48.1 ± 11.9 y; 34 controls, 21F, 43.3 ± 10.1 y)	TN pain: 6.31 ± 1.15, 6.02 ± 4.35 y/None	Structural MRI Functional MRI	TN: ↓GM in bilateral amygdala, PAG, R insula; rsFC: ↓ L amygdala, L thalamus/putamen/DLPFC, ↑R amygdala, R PFC; clinical correlations: L amygdala–DLPFC–pain duration (neg), HAMD/HAMA-R amygdala–R PFC (pos); post treatment—rsFC normalized.
[73] Cohort observational study	12 TN, 8F, 68.9 y); 4 controls, 2F, 63.3 ± 4.4 y	TN pain 9.25 ± 0.97/radiofrequency	Structural MRI Diffusion MRI	VAS: ↓ at 1 mo, slight ↑ at 6 mo, ↓ at 12 mo; DTI (REZ + nuclear zone)—side differences in FA/diffusivity, nuclear FA ↓ at 1 mo, ↑ 1–6 mo; GMV asymmetry—affected side ↓ pre treatment, absent 1 mo, present 6 mo, absent 12 mo; DTI–GMV correlations—widespread pre treatment, region-specific 1–12 mo post
[87] Case–control observational study	48 TN, 22F, 62.5 ± 5.7 y; 35 controls, 62.4 ± 5.6 y	TN duration: 2.65 ± 0.39 y/None	Structural MRI Diffusion MRI Functional MRI	PTN rsFC: ↑ Spinal cord/thalamus–postcentral & midfrontal gyri, ↓ R supramarginal; clinical correlations—↑ FC with postcentral, duration (pos), ↓ FC with R supramarginal, duration (neg).
[98] Case–control observational study	TN: R-sided n = 23 (15F, 47 ± 12 y, 23–67), L-sided n = 14 (9F, 55 ± 10 y, 36–70); HCs: n = 28 (47 ± 12 y R-TN match; 53 ± 10 y L-TN match)	Chronic TN pain/None	Structural MRI Diffusion MRI	TN: Middle cingulum ↓ FA, ↑ MD/RD bilaterally (L-TN > R-TN); Posterior cingulum L-TN stronger FA & AD ↓ L hemisphere vs. R-TN; MFB/VTA: dMRI differences, L-TN ↓ MD/RD R side vs. R-TN, additional L MFB–VTA alterations in R-TN vs. HCs; GMV: bilateral ↓ PCC, NAc, VD, hippocampus; no change rostral/mid ACC or isthmus
[89] Case–control observational study	TN: n = 46 (31F, 57.6 ± 10.2 y); HCs: n = 35 (18F, 54.2 ± 8.5 y)	TN duration: 4.21± 4.96 y/None	Structural MRI Diffusion MRI	TN patients exhibited lower FA and higher MD/RD, with strongest effects in the splenium of the corpus callosum, bilateral anterior corona radiata, external capsule, tapetum, and fornix.

Legend: ACC: Anterior Cingulate Cortex; BNI: Barrow Neurological Institute Pain Intensity Score; CPI: Current Pain Intensity; CRF: Continuous Radiofrequency; DLPFC: Dorsolateral Prefrontal Cortex; DMN: Default Mode Network; dACC: Dorsal Anterior Cingulate Cortex; FA: Fractional Anisotropy; GMV: Gray Matter Volume; HADS: Hospital Anxiety and Depression Scale; HCs: Healthy Controls; IPL: Inferior Parietal Lobule; ITG: Inferior Temporal Gyrus; LGI: Local Gyrification Index; MFB: Medial Forebrain Bundle; MD: Mean Diffusivity; mPFC: Medial Prefrontal Cortex; NAc: Nucleus Accumbens; PAG: Periaqueductal Gray; PIDP: Persistent Idiopathic Dentoalveolar Pain; PRF: Pulsed Radiofrequency; PTN: Post-Traumatic Trigeminal Neuropathy; PCC: Posterior Cingulate Cortex; REZ: Root Entry Zone; rsFC: Resting-State Functional Connectivity; SF-MPQ: Short-Form McGill Pain Questionnaire; S1: Primary Somatosensory Cortex; S2: Secondary Somatosensory Cortex; TN: Trigeminal Neuralgia; VAS: Visual Analog Scale; VD: Ventral Diencephalon; VTA: Ventral Tegmental Area.

**Table A3:** Included Human MRI Studies of Temporomandibular Disorders.

Reference	Participants	Intervention/Pain	Image	Main MRI Findings
[46] Case–control observational study	11 TMD, 10F, 25.8 ± 2.3 y; 11 controls, 10F, 24.8 ± 1.2 y	Chronic myofascial TMD: mean pain 3.8 ± 2.2/10; duration 0.5–7 y	Structural MRI	TMD MRS: Left insula Glu ↓ post test, Glx pre test ↑ with pain; NAA & Cho ↑, NAA pain duration; Right insula Gln ↑ vs. left and HCs, Gln pain scores.
[40] Case–control observational study	14 TMJ synovitis, 12F, 33.7 ± 13.2 y; 14 controls, 7F, 23.7 ± 0.9 y/maximal clenching task	TMJ biting pain: contralateral pain (n = 8) vs. ipsilateral pain (n = 6).	Structural MRI Functional MRI	ACC with biting pain (absent in HCs); reduced activation in IFG, precentral, STG, MFG vs. HCs; task-specific IFG/Broca’s activation during unilateral clenching, stronger left hemisphere for left clench; activation patterns modulated by pain laterality
[92] Case–control observational study	9F TMD myofascial; 9 matched controls	Chronic myofascial TMD pain. Average duration ≈ 2.5 years	Structural MRI	GM ↓: L ACC, R PCC, R IC, L IFG, bilateral STG → R MTG (R STG/MTG ~ pain duration); WM ↓: bilateral MFG/SFG, L precuneus, L MFG/IFG; WM ↑: posterior STG bilaterally → R SMG
[91] Case–control observational study	13F TMD, 28.7 ± 7.6 y; 12F controls, 28.8 ± 7.9 y/Flutter stimulation	Chronic TMD: mean pain 2.4/10; perceived flutter 32.0 ± 15.4 vs. HCs 19.2 ± 12.5	Structural MRI Functional MRI	TMD showed greater posterior insula, ACC, and contralateral amygdala activation; controls showed stronger anterior insula activation.
[71] Case–control observational study	47F TMD, 28.8 ± 9.7 y; 60F controls, 37.3 ± 12.0 y; 57 facial pain, 43F, 47.6 ± 16.5 y, 381 matched controls 287F, 47.6 ± 13.3 y	Chronic TMD/facial pain: Clinical: VAS rest 15.7 ± 18.1, TMJ movement 23.9 ± 22.4; Population cohort: NRS rest 3.6 ± 1.4	Structural MRI	One significant cluster from ACC to mPFC; highest effect in anterior medial ACC (MNI coordinate), showing smaller GMV in patients.

Legend: ACC: anterior cingulate cortex; NRS: numerical rating scale; TMJ: temporomandibular joint; TMD: temporomandibular disorder; VAS: visual analog scale.
